# Dynamic changes in carbohydrate components and the bacterial community during the ensiling of wilted and unwilted sweet sorghum

**DOI:** 10.3389/fmicb.2024.1452798

**Published:** 2024-08-19

**Authors:** Zhiqiang Sun, Yiting Liu, Fangcai Ji, Shuangye Li, Lei Wang, Zhenming Zhou, Zhe Wu, Zhu Yu

**Affiliations:** ^1^College of Grassland Science and Technology, China Agricultural University, Beijing, China; ^2^State Key Laboratory of Animal Nutrition, College of Animal Science and Technology, China Agricultural University, Beijing, China

**Keywords:** sweet sorghum, microbial community, carbohydrate components, biofuel, ensiling

## Abstract

Sweet sorghum can be used to produce a substantial quantity of biofuel due to its high biological yield and high carbohydrate content. In this study, we investigated the dynamic changes in fermentation characteristics, carbohydrate components, and the bacterial community during the ensiling of wilted and unwilted sweet sorghum. The results revealed a rapid fermentation pattern and high-quality fermentation quality in wilted and unwilted sweet sorghum, wherein lactic acid, and acetic acid accumulated and stabilized during the initial 9 days of ensiling, with the pH values less than 4.2, until 60 days of ensiling. We found that the ensiling of sweet sorghum involved the degradation (5% ~ 10%) of neutral detergent fiber (NDF) and hemicellulose and that the degradation of NDF fit a first-order exponential decay model. A shift in dominance from *Lactococcus* to *Lactobacillus* occurred before the first 9 days of ensiling, and the abundance of *Lactobacillus* (*r* = −0.68, *p* < 0.001) was negatively correlated with the NDF content. The relative abundances of *Lactobacillus* in wilted and unwilted sweet sorghum after ensiling for 60 days were 76.30 and 93.49%, respectively, and relatively high fermentation quality was obtained. In summary, ensiling is proposed as a biological pretreatment for sweet sorghum for subsequent biofuel production, and unlike other materials, sweet sorghum quickly achieves good fermentation quality and has great potential for bioresource production.

## 1 Introduction

The production of bioresources is under great pressure due to global climate change and the continuous emergence of issues such as urbanization and water scarcity (Getachew et al., 2016). Photosynthetic green plants are the primary source of biomass used to produce biofuels, which are renewable and sustainable energy sources (Chandel and Singh, 2011). In general, plants that have more carbohydrates and less lignin generally have the benefit of being able to produce significant amounts of biofuel (Mathur et al., 2017). Sweet sorghum (*Sorghum bicolor* L. Moench) is a C4 herbaceous annual grass that has high photosynthetic efficiency. It can grow in semiarid and highly saline areas since it adapts well to environments with little rain, extreme temperatures, and poor soil conditions (Amer et al., 2012). Moreover, sweet sorghum has comparatively high energy and carbohydrate contents. After the required processing, the accessible holocellulose (HoC) in the biomass may be readily used to produce biofuel (Velmurugan et al., 2020). Currently, maize, sugarcane, and soybean are the crop plants most widely used as sources of biofuel (Mathur et al., 2017). However, compared to food crops such as maize, sugarcane, and soybeans, the choice to use sweet sorghum as fuel avoids the conflict between food and fuel (Ren et al., 2021). Consequently, sweet sorghum appears to be an attractive feedstock for the manufacture of biofuel (Zhao et al., 2009; Mathur et al., 2017). However, owing to its short harvest periods and seasonality, sweet sorghum requires appropriate preservation after widespread harvest to guarantee a steady supply throughout the year for subsequent biofuel production (Herrmann et al., 2015).

Ensiling is an effective pretreatment method for ensuring a continuous supply of feedstock because it conserves more plant energy and increases the effectiveness of enzymatic hydrolysis (Li et al., 2018; Zhao et al., 2018). During the anaerobic fermentation process, water-soluble carbohydrates (WSCs) are utilized by microorganisms and converted to organic acids, which reduce the pH to levels, thereby inhibiting the activity of microorganisms such as *Clostridium*, *Shigella*, and *Salmonella* spp. (Janke et al., 2019). At the same time, ensiling can be used as a beneficial measure to change the structure and composition of fibers and facilitate fiber degradation (Zhao et al., 2018; Wu et al., 2020). Thus, ensiling treatments offer new perspectives for the efficient conversion of biomass (Ambye-Jensen et al., 2013). Previous studies have shown that inadequate concentrations of organic acids during the ensiling process can lead to a loss of organic matter ranging from 18 to 35% (Kreuger et al., 2011). This loss is caused by butyrate fermentation, which produces H_2_ and CO_2_, and is carried out by unfavorable *Clostridia*.

Sweet sorghum is a plant with excellent ensiling properties because of its low buffering capacity, sufficient levels of soluble carbohydrates, and high moisture and structural carbohydrate contents (Ren et al., 2021). In general, materials ensiled at high moisture levels are particularly susceptible to butyrate fermentation, which reduces fermentation quality and leads to greater organic matter losses (Li et al., 2020). However, our previous research has shown that good silage quality can be achieved without the addition of additives to sweet sorghum, even at high moisture contents (Sun et al., 2022). Previous studies have also shown that sweet sorghum silage fermentation quality is not positively affected by microbial or enzyme inoculants (Diepersloot et al., 2021). During the production process, after sweet sorghum is harvested, some raw materials cannot be pretreated in a timely manner, and it is possible that some may wilt in the field. The properties of the raw material, especially the moisture and microbial content, are often affected by the wilting treatment, which may impact the overall ensiling process and in turn affect the changes in microbial communities and carbohydrate components (Muck, 1988). However, to our knowledge, there is limited research on the changes in carbohydrate components and microbial communities during the ensiling of sweet sorghum.

Thus, in this study, we investigated the dynamic changes in carbohydrate components and microbial communities of wilted and unwilted sweet sorghum during ensiling pretreatment, as well as the impact of ensiling processes on the fiber degradation kinetics of sweet sorghum. The results of this study will contribute new information and insights regarding the efficient pretreatment of sweet sorghum as a feedstock for biofuel production.

## 2 Materials and methods

### 2.1 Experiment site and material preparation

Sweet sorghum plants (*Sorghum bicolor* L. F438) were harvested at the heading stage in Wuzhong, Ningxia Hui Autonomous Region, China (N 37°43′, E 106°04′, elevation 1,240 m). The experimental area is dry year-round with little rain, and there is a large temperature difference between day and night. This environment is typically a temperate continental climate, and the amount of rainfall is relatively low, with an evaporation rate 9 times greater than that of rainfall under limited irrigation conditions, resulting in poor soil tolerance. A portion of the test materials was collected immediately after harvest, the moisture contents was 760 g/kg, and additional materials were obtained after 48 h of wilting in the field, the moisture contents was 727 g/kg. The wilted and unwilted materials were shredded into approximately 2 cm pieces using a small plant chopper. Approximately 300 g of fresh material was collected for further analysis. Silage preparation was performed as follows: the wilted and unwilted materials were roughly and randomly split into multiple equal parts, and approximately 800 g samples of the shredded plants were put in vacuum bags (type; 35 × 45 cm; Deli Group Co., Ltd., Zhejiang, China). The 42 bags (2 treatments ×7 days × 3 replicates) were vacuum-sealed and kept in a room at approximately 20°C. Three bags per treatment were opened after the initial (3d, 6d, 9d), intermediate (14d, 21d, 30d) and late (60d) fermentations. The moisture content of the materials was measured dynamically throughout the ensiling process, and the final moisture contents of unwilted and wilted sweet sorghum was 758 g/kg and 727 g/kg, respectively. In our study, there was no significant change in the moisture content of the biomass material (Supplementary Figure S1), nor was there a significant change in the biomass weight. Then the fermentation characteristics, carbohydrate components, and microbial communities of the preensiled materials and silage samples were examined.

### 2.2 Fermentation characteristics analyses

Our prior study methodology is described to in this chapter (Sun et al., 2022). Briefly, the sample (20 g) in each bag was mixed with sterilized distilled water (180 mL). The mixture was homogenized for 1 min using a plant juicer extractor with 30 pulses (2 s each). The resulting mixture was then passed through cheesecloth and one layer of qualitative filter paper. Glass-electrode pH meters were used to determine the pH of the silage. The filtrate was stored at −20°C for subsequent analysis of organic acids such as lactic acid (LA), acetic acid (AA) propionic acid (PA), and butyric acid (BA) by high-performance liquid chromatography (HPLC; Shimadzu, Tokyo, Japan). The concentration of ammonia-nitrogen (AN) was determined using the phenol and sodium hypochlorite method (Broderick and Kang, 1980).

### 2.3 Chemical composition and carbohydrate component analyses

Preensiled materials and silage samples (300 g) were collected for chemical composition analysis. The well-mixed samples were dried in a blast oven (65°C) for 48 h to achieve a constant weight, which was used to calculate the dry matter (DM) content. The dried samples were milled to a size of 1 mm using a plant grinder. The crushed samples were then collected for examination of their chemical composition and carbohydrate components. The anthrone-sulfuric acid method was applied to determine the WSC content (McDonald and Henderson, 1964). The contents of neutral detergent fiber (NDF) and acid detergent fiber (ADF) were analyzed using an automatic fiber analyzer (Ankom 2000i full; Ankom Tech Co., Macedon, NY, United States) in accordance with the procedures outlined by Van Soest et al. (1991). The hemicellulose (HC) content was estimated as the NDF value minus the ADF value. The acid detergent lignin (ADL) content was determined according to the method described by Van Soest (1973). Similarly, the cellulose (CL) content was estimated as the ADF value minus the ADL value, and the HoC content was estimated as the sum of the HC value and CL value. Using an automated Kjeldahl apparatus (FOSS KJ2300, United States), the nitrogen (N) concentration was multiplied by 6.25 to calculate the crude protein (CP) content. The starch content was obtained using the method described by Rose et al. (1991).

### 2.4 Fiber degradation kinetics analyses

The NDF, ADF, HC, and CL data were fitted to the following exponential decay model (Equation 1) to characterize the fiber degradation properties (DePeters et al., 1997; Zhao et al., 2018):


(1) $$y=y_{0}+a*\exp\;\left(-\mathrm{bt}\right)\;$$

Among these are: y, the total amount of undegraded material at time t; y_0_, the undegraded portion fraction after 60 days of ensiling; a, the degradable fraction; b, the percentage disappearance rate (day^−1^) of a; t, the number of ensiling days; and e, 2.7183.

### 2.5 Bacterial community analyses

The total microbial DNA extraction of the sweet sorghum silage samples was performed according to the method described by Jia et al. (2021). 20 g samples was mixed with 80 mL of sterile water, and stirred at 120 rpm and 4°C for 2 h. The samples were filtered through two layers of sterile gauze and then centrifuged at 10,000 × g for 15 min at 4°C. The pellet was washed three times in sterile water and used for total genomic DNA extraction. The integrity of the total DNA was subsequently confirmed via 1% agarose gel electrophoresis. Then, utilizing a PCR thermocycler, the V3–V4 hypervariable portions of the bacterial 16S rRNA gene were amplified with the primers 338F (5′- ACTCCTACGGGAGGCAGCAG-3′) and 806R (5’-GGACTACHVGGGTWTCTAAT-3′). The Illumina PE300 platform for sequencing (Illumina, San Diego, United States) was used to purify and quantify the resulting PCR products. Finally, the raw sequences were further analyzed according to the procedure described by Jia et al. (2021).

### 2.6 Statistical analysis

The data were collated using Excel 2020. The effects of wilting treatment and ensiling duration on the fermentation characteristics, carbohydrate components, and the relative abundance of the bacterial communities were analyzed by IBM SPSS 18.0 software. ANOVAs were used to evaluate the experimental results. Multiple comparisons were conducted using Duncan’s test, with differences deemed statistically significant at *p* < 0.05. A t test was used to compare the differences in fermentation characteristics and carbohydrate components between the wilted and unwilted groups at the same ensiling time, and the differences were considered statistically significant at *p* < 0.05. Using a nonlinear regression (NLIN) process in SPSS, an iterative least squares method was used to estimate the fiber degradation kinetic parameters y_0_, a, and b. The correlation of the bacterial composition and fermentation characteristics/carbohydrate components was examined using Spearman correlation. A free online platform^1^ was used to analyze the high-throughput sequencing data.

## 3 Results and discussion

### 3.1 Chemical composition of sweet sorghum prior to ensiling

The chemical compositions of wilted and unwilted sweet sorghum prior to ensiling are presented in Table 1. The DM contents of wilted and unwilted sweet sorghum were 273.07 and 239.41 g/kg, respectively. The WSC levels (233.15 and 236.36 g/kg DM in wilted and unwilted sweet sorghum, respectively) were greater than the threshold value (60 to 70 g/kg DM) for well-fermented silage (Smith, 1962). These results were similar to our previous result (210.47 g/kg DM) (Sun et al., 2022). The CP, NDF, starch, etc., play decisive roles in the evaluation of plant characteristics (Sun et al., 2022). In this study, the CP content of wilted and unwilted sweet sorghum was approximately 60 g/kg DM, and the NDF content was approximately 520 g/kg DM. In previous studies, the starch content in sweet sorghum was 40 ~ 100 g/kg DM (Sun et al., 2022), while in the present study, the starch contents of wilted and unwilted sweet sorghum were 35.00 and 49.04 g/kg, respectively. This difference may be related to the variety and growing environment of sweet sorghum.

**Table 1:** Chemical composition of wilted and unwilted sweet sorghum prior to ensiling.

Item	Value ± SD (*n* = 3)	
	Unwilted	Wilted
Dry matter (g/kg)	239.41 ± 1.21	273.07 ± 2.73
Water-soluble carbohydrate (g/kg DM)	236.36 ± 13.73	233.15 ± 2.61
Neutral detergent fiber (g/kg DM)	528.88 ± 6.11	527.80 ± 6.56
Acid detergent fiber (g/kg DM)	313.15 ± 2.37	308.18 ± 3.52
Hemicellulose (g/kg DM)	215.72 ± 4.26	219.62 ± 3.13
Cellulose (g/kg DM)	282.81 ± 1.91	278.80 ± 3.87
Acid detergent lignin (g/kg DM)	23.51 ± 0.45	21.13 ± 0.99
Crude protein (g/kg DM)	61.13 ± 1.36	57.22 ± 0.87
Starch (g/kg DM)	49.04 ± 4.77	35.00 ± 3.34

DM, dry matter; SD, standard deviation.

### 3.2 Changes in the fermentation characteristics of sweet sorghum during ensiling

**Figure 1:**
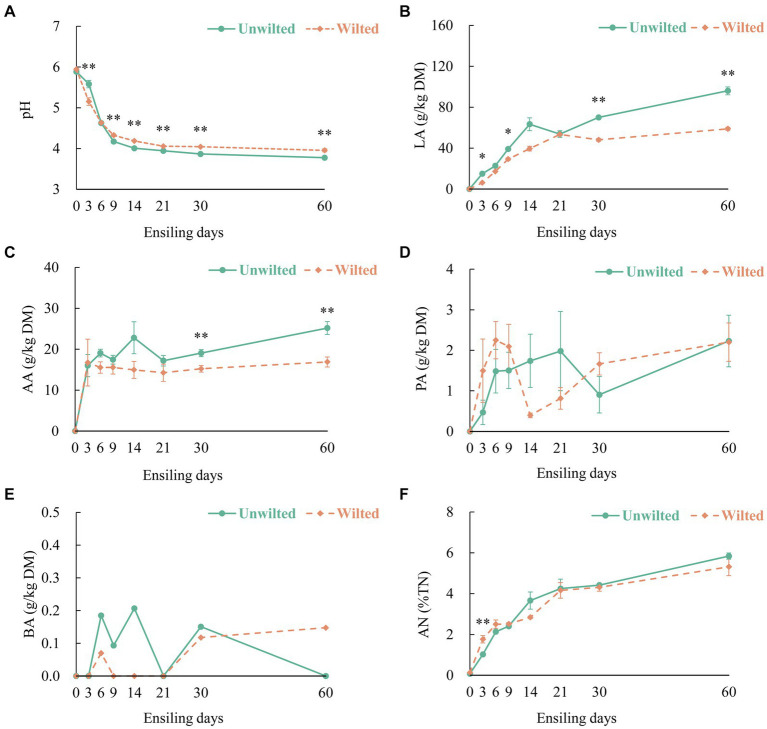
The dynamic changes in **(A)** pH, **(B)** lactic acid (LA), **(C)** acetic acid (AA), **(D)** propionic acid (PA), **(E)** butyric acid (BA), and **(F)** ammonia- nitrogen (AN) in wilted and unwilted sweet sorghum after 3, 6, 9, 14, 21, 30 and 60 days of ensiling. DM, dry matter; TN, total nitrogen; Asterisks indicate significant differences between different treatments on the same ensiling days (*, significant at *p* < 0.05, and **, significant at *p* < 0.01).

The dynamic changes in the pH, organic acid content, and AN in wilted and unwilted sweet sorghum during the ensiling are shown in Figures 1A–F. The pH is an essential parameter for evaluating fermentation performance, especially for silages with high moisture content (Wang Y. et al., 2018). In this study, the increase in LA and AA accumulated and stabilized during the initial 9 days of ensiling, with the pH values less than 4.2, until 60 days of ensiling. With increasing ensiling time, the LA content of wilted and unwilted sweet sorghum silage continuously increased, exhibiting a pattern of rapid increase in the initial phase of ensiling and a slow increase in the later phase of ensiling. Similarly, the AA content also exhibited the same trend. This pattern was in accordance with the results reported by Ni et al. (2017) and Wang et al. (2019a). Moreover, after 30 days of ensiling, the LA and AA contents of the unwilted sweet sorghum silage were greater than those of the wilted silage (*p* < 0.01). This difference may be due to the greater abundance of Lactobacillus in the unwilted sweet sorghum silage. A high WSC content in sweet sorghum can rapidly boost the growth of lactic acid bacteria and initiate LA fermentation in the early stages, favoring the dominance of Lactobacillus spp., which inhibits the growth of other hazardous microbes (McDonald et al., 1991; Wang et al., 2020). This finding is in line with our earlier research, which showed that even under high moisture conditions, anaerobic fermentation of sweet sorghum could achieve relatively high levels of LA and relatively low pH (Sun et al., 2022).

It is commonly assumed that PA and BA represent inefficient or secondary fermentation (Zhao et al., 2022). In this experiment, as the ensiling time increased, the PA content in the wilted and unwilted sweet sorghum silages gradually increased. After 60 days of ensiling, the PA content was approximately 2 g/kg DM, which is consistent with previous research results (Zhao et al., 2022). The BA level is an important factor that affects quality, and the BA content of high-quality fermented silage is less than 1 g/kg DM. In this study, extremely low amounts of BA (less than 0.3 g/kg DM) were detected in the wilted and unwilted silage samples during the whole ensiling process. Another important factor in assessing silage quality is the AN level, which indicates the degree of CP degradation in the material (Wang S. et al., 2018). In the present study, the AN concentration in wilted and unwilted sweet sorghum silage increased with prolonged ensiling time, and the AN content at 60 days of ensiling was approximately 6% of the total nitrogen (TN), below the threshold of approximately 10%. This could be ascribed to the lower pH possibly inhibiting the proliferation and proteolytic ability of microbes such as *Clostridia* (Arriola et al., 2011).

### 3.3 Changes in the carbohydrate components of sweet sorghum during ensiling

**Figure 2:**
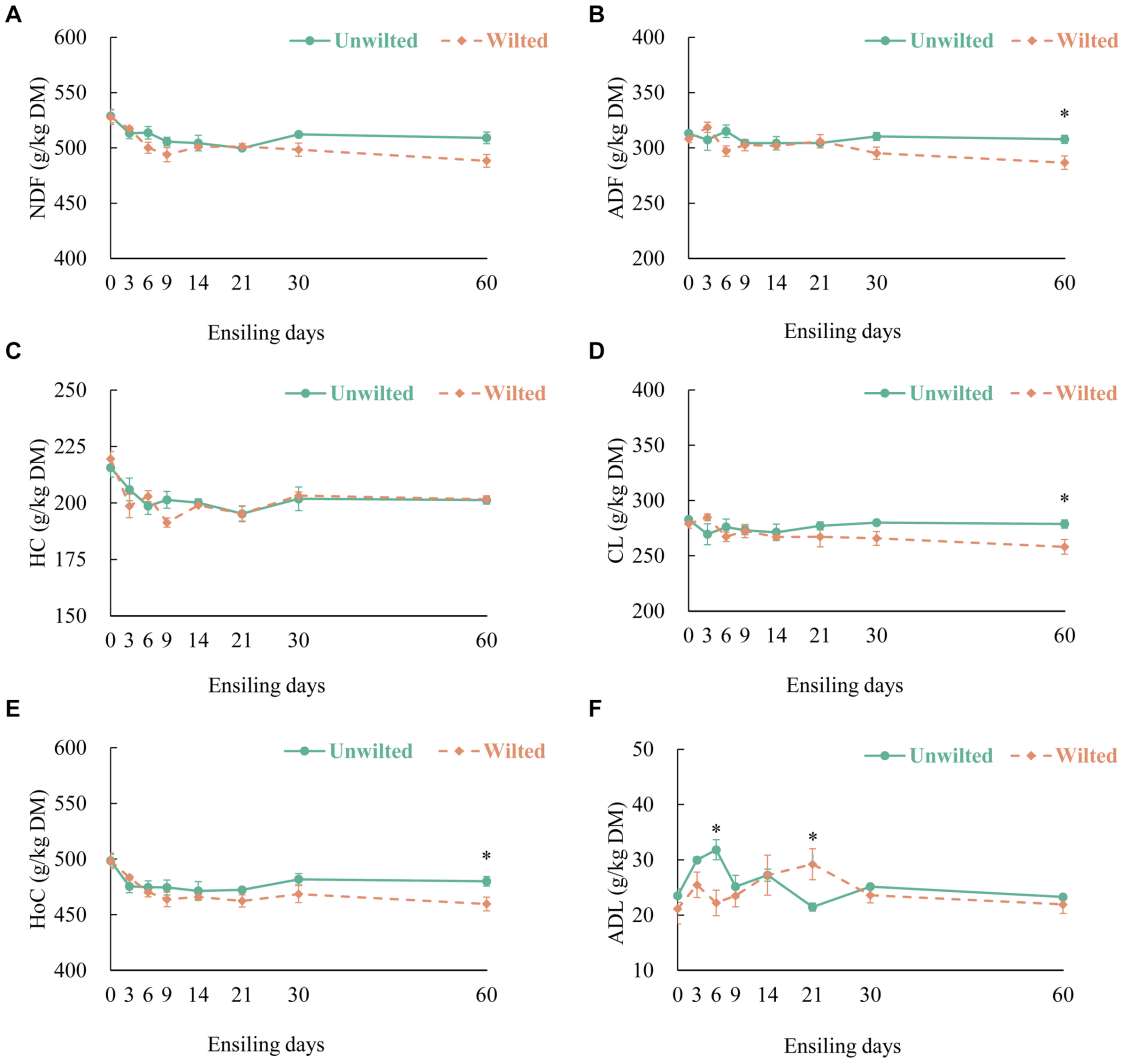
The dynamic changes in **(A)** neutral detergent fiber (NDF), **(B)** acid detergent fiber (ADF), **(C)** hemicellulose (HC), **(D)** cellulose (CL), **(E)** holocellulose (Hoc), and **(F)** acid detergent lignin (ADL) contents in wilted and unwilted sweet sorghum after 3, 6, 9, 14, 21, 30 and 60 days of ensiling. DM, dry matter. Asterisks indicate significant differences between different treatments on the same days of ensiling (*, significant at *p* < 0.05, and **, significant at *p* < 0.01).

The dynamic changes in the Figure 2A (NDF), Figure 2B (ADF), Figure 2C (HC), Figure 2D (CL), Figure 2E (HoC) and Figure 2F (ADL) contents in wilted and unwilted sweet sorghum during the ensiling. With increasing ensiling time, the NDF, HC, and HoC contents in the wilted and unwilted sweet sorghum silages decreased to varying degrees, the ADF and CL contents in the unwilted sweet sorghum silage remained basically unchanged, and the ADF and CL contents in the wilted sweet sorghum silage tended to decrease during the later stage of ensiling. The degradation mechanism of structural carbohydrates observed in the study could be explained by enzymatic action, acidolysis and microbial activity (Dewar et al., 1963). Unlike other materials, sweet sorghum has a higher content of WSCs and a lower buffering capacity. After the formation of an anaerobic environment, the microbial fermentation process intensifies, and the acid production ability of the microbial mixture becomes stronger, which may lead to a certain degree of degradation of structural carbohydrates. Zhao et al. (2018) indicated that as ensiling time increased, cell wall degradation occurred, which could be attributed to the low pH of the silage promoting the acid hydrolysis of HC. Several studies have shown that the degradation of HC during ensiling may be related to the presence of bacterial genera such as *Betabacterium* and *Pentoaceticum* (*Lactobacillus brevis*) in silage (Dewar et al., 1963; Xie et al., 2022). In this study, we did not evaluate the products of HC degradation. However, there is evidence that at least some pentose sugars are end products of HC degradation during ensiling, as the main residual sugars in silage extracts are galactose and xylose (Dewar et al., 1963). In our study, there was no change in the ADL content in wilted or unwilted sweet sorghum silage with increasing ensiling time, and similar results have been obtained in previous studies (Tao et al., 2011). Therefore, enzymatic action, acidolysis and microbial activity during ensiling lead to the degradation of components such as CL and HL in the sweet sorghum cell wall, which reduces the stiffness and integrity of the cell wall. The WSC content in unwilted and wilted sweet sorghum silage was decreased, mainly due to the consumption of WSC by lactic acid bacteria and other microorganisms for fermentation (Hu et al., 2009) (Supplementary Figure S2A). Similarly, the starch content was also decreased (Supplementary Figure S2B). In our study, we did not evaluate the changes in the protein structure and physical structure of sweet sorghum during ensiling. However, previous studies have shown that the protein structure and physical structure of biomass materials underwent changes after ensiling, which contributed to the enhanced enzymatic hydrolysis and consequently the release of reducing sugars (Zhao et al., 2018).

### 3.4 Kinetic degradation of structural carbohydrates

**Figure 3:**
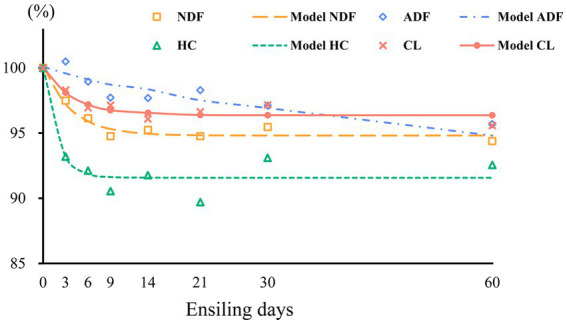
Degradation curves of neutral detergent fiber (NDF), acid detergent fiber (ADF), hemicellulose (HC), and cellulose (CL) in wilted and unwilted sweet sorghum silage; the results are expressed as percentages of the raw material.

The measured structural carbohydrate contents were fitted using a first-order exponential decay model, and the kinetic parameters and related fit curves are shown in Table 2 and Figure 3, respectively. We found that the correlation coefficient (R^2^ value) of the NDF degradation curve was 0.957, which indicates that the degree of NDF degradation in sweet sorghum silage can be well predicted using a first-order exponential decay model. Degradation of NDF occurred mainly during the initial 14 days of ensiling, suggesting that structural carbohydrates are more readily degraded during the early phases of ensiling. Similar results were also found in a previous study, in which the combination of *Lactobacillus plantarum* and cellulase increased the breakdown of structural carbohydrates in forage silage (Zhao et al., 2018). ADF showed slight degradation, and the degradation rate was 0.04, which could be attributed to continued acidolysis. The large reduction in NDF was attributed to the high breakdown of HC, which is consistent with the finding that HC is readily hydrolyzed (McDonald et al., 1991). Furthermore, the protective layers of lignin and HC inhibited the breakdown of CL, which could account for the strong NDF and weak ADF degradability of sweet sorghum. In our study, about 8% HC and other minor carbohydrate components were degraded during ensiling of sweet sorghum, which may have a positive impact on the biofuel production process. In general, the role of pretreatment is not only to degrade specific components of the raw material, but also to change the physical and chemical properties of the raw material, remove impurities and create more favourable conditions for subsequent bioconversion processes (Poddar et al., 2022). Although the degradation of carbohydrates during the ensiling process optimises the quality and characteristics of the feedstock required for biofuel production, but more thorough and precise pretreatment operations may be required to ensure the efficiency of the fermentation process as well as the purity and yield of the product. Therefore, this degradation effect during the ensiling process can be used as part of the reference for the improving of the production process, but it cannot be relied upon alone to completely avoid the high energy and expensive problems of the pretreatment protocols.

**Table 2:** Degradation kinetic parameters of fiber during the ensiling of sweet sorghum and fitted to a first-order exponential decay model (y = y_0_ + a * exp. (−bt)).

Item^a^	y_0_	a	b	R^2^
NDF	94.81	5.26	0.25	0.957
ADF	95.55	4.54	0.04	0.821
HC	91.57	8.43	0.55	0.882
CL	96.37	3.67	0.25	0.868

a NDF, neutral detergent fiber; ADF, acid detergent fiber; HC, hemicellulose; CL cellulose.

### 3.5 Changes in the microbial community of sweet sorghum during ensiling

As shown in Table 3. The number of recovered reads per sample ranged from 41,072 to 54,270. All the coverage values were greater than 0.99, which means that the majority of the bacteria were detected. For further analysis, 1731 operational taxonomic units (OTUs) were clustered at the 97% similarity level. The Chao1 index and number of OTUs decreased with increasing ensiling time, and at 0 days of ensiling, the Chao1 index was greater than that at other ensiling days (*p* < 0.01), indicating that community richness decreased over time and that bacterial community diversity was highest in preensiled sweet sorghum.

**Table 3:** Alpha diversity of bacteria in wilted and unwilted sweet sorghum during ensiling.

Treatments	Ensiling days	Reads	OTUs	Chao1	Coverage	Shannon
Unwilted	0 d	54279a	291a	338.75a	0.998	2.8
	3 d	53244ab	135c	189.88b	0.999	2.15
	9 d	45959ab	153bc	200.51b	0.999	2.82
	21 d	43693b	162b	208.21b	0.999	3.01
	60 d	42850b	104d	227.27b	0.998	2.23
Wilted	0 d	47,353	277a	362.52a	0.997	3.17ab
	3 d	42,281	136b	206.14b	0.999	2.88b
	9 d	41,072	152b	184.92bc	0.999	3.28a
	21 d	47,907	169b	200.22b	0.999	3.32a
	60 d	41,998	152b	151.93c	0.999	2.13c
SEM^a^		2,997	10	22.61	0.0001	0.21
*p*-value^b^	WT	0.054	0.230	0.420	0.133	0.017
	ED	0.073	<0.001	<0.001	<0.001	0.001
	WT × ED	0.153	0.082	0.241	0.128	0.446

Means within the same column (a-c) with lowercase letters are significantly different (*p* < 0.05).

a SEM, standard error of means.

b WT, wilting treatment; ED, ensiling days; WT×ED, the interaction between wilting treatment and ensiling days.

**Figure 4:**
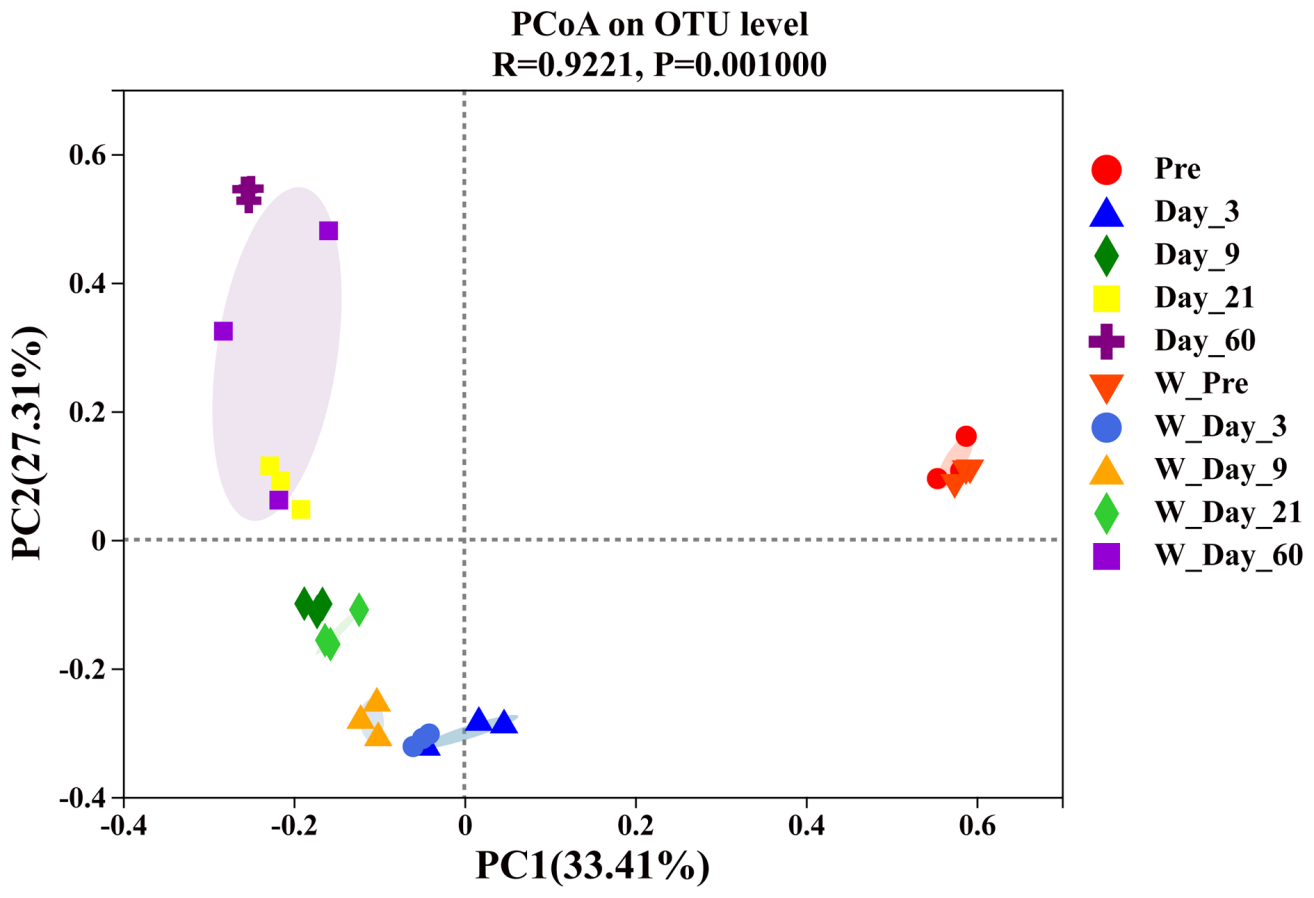
Principal coordinate analysis (PCoA) of β_diversity in wilted and unwilted sweet sorghum during ensiling. Pre, preensiled materials of unwilted sweet sorghum; Day_3, Day_9, Day_21 and Day_60, unwilted sweet sorghum after 3, 9, 21, and 60 days of ensiling; W_Pre, preensiled materials of wilted sweet sorghum; W_Day_3, W_Day_9, W_Day_21 and W_Day_60, wilted sweet sorghum after 3, 9, 21, and 60 days of ensiling.

The β-diversity analysis was carried out to determine the similarities or differences in microbial community compositions between different groups or samples. As shown in Figure 4, principal coordinate analysis (PCoA) revealed the variation in the microbial community. The samples from different ensiling times were separated from each other, which suggested that ensiling time had an impact on the microbial community composition. At the same ensiling time, the samples from the unwilted and wilted groups were closely clustered, which showed that these microbial communities had relatively similar compositions. The lower variability may be explained by the rapid decrease in pH in wilted and unwilted sweet sorghum after the formation of an anaerobic environment, which may limit microbial proliferation and consequently reduce microbial growth and diversity (Wang et al., 2019b).

**Figure 5:**
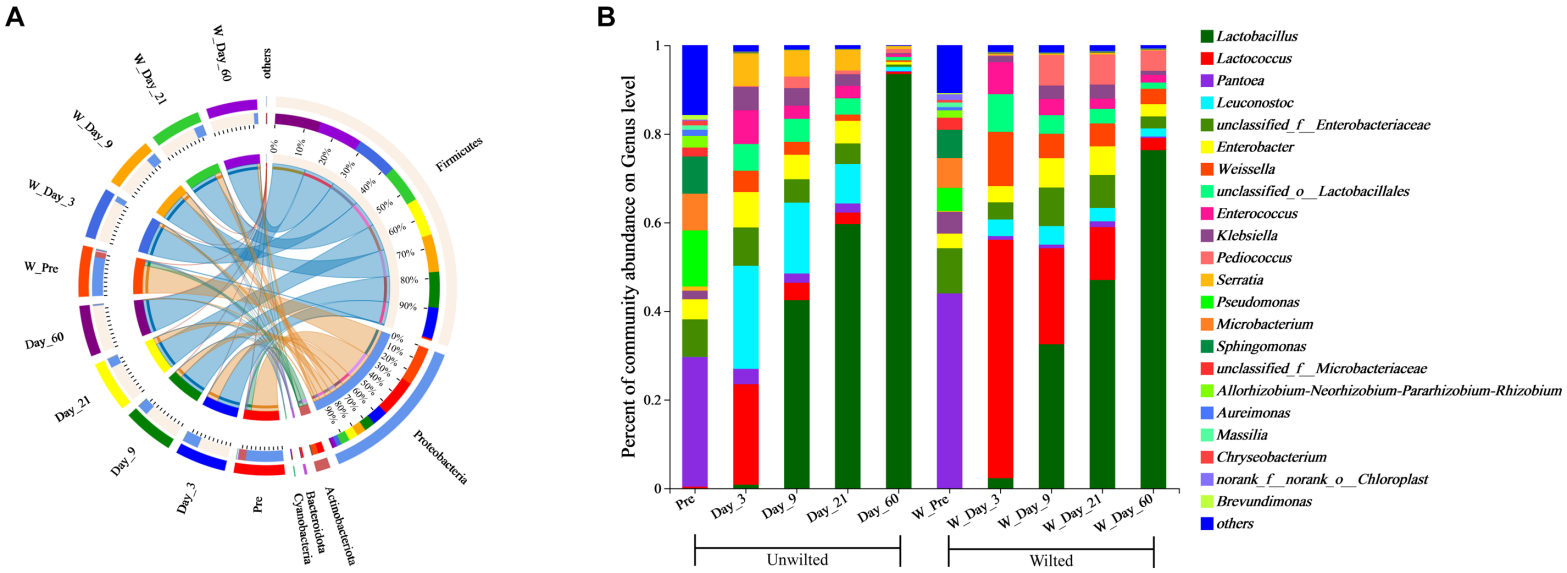
Bacterial community composition at the phylum **(A)** and genus **(B)** levels in wilted and unwilted sweet sorghum during ensiling. Pre, preensiled materials of unwilted sweet sorghum; Day_3, Day_9, Day_21 and Day_60, unwilted sweet sorghum after 3, 9, 21, and 60 days of ensiling; W_Pre, pPreensiled materials of wilted sweet sorghum; W_Day_3, W_Day_9, W_Day_21 and W_Day_60, wilted sweet sorghum after 3, 9, 21, and 60 days of ensiling.

A Circos plot showing differences in the relative abundance of bacterial community at the phylum level was prepared as shown in Figure 5A. The results indicated that Proteobacteria was the dominant phylum in preensiled sweet sorghum, and the relative abundance of Proteobacteria in wilted and unwilted sweet sorghum was approximately 80%. Actinobacteriota and Bacteroidota were two other main phyla in the preensiled sweet sorghum. Similarly, these phyla were also found in other materials, and their abundances were related to plant type (Nazar et al., 2020; Xie et al., 2022). The changes in bacterial phyla during the ensiling process of wilted and unwilted sweet sorghum showed the same regular pattern. With extended ensiling time, the relative abundance of Proteobacteria gradually decreased, while that of Firmicutes gradually increased. After 3 days of ensiling, Firmicutes emerged as the dominant phylum, with a relative abundance greater than 50%. After 60 days of ensiling, Firmicutes was the overwhelmingly dominant phylum, with a relative abundance greater than 90%. Zhao et al. (2023) demonstrated that Proteobacteria dominated the microbiota of fresh sweet sorghum, whereas Firmicutes was the dominant phylum in all the microbial communities after anaerobic fermentation; these findings are consistent with our research results. In fact, the succession from Proteobacteria to Firmicutes in anaerobic environments has often been documented (Yang et al., 2019; Xie et al., 2022). The ensiling process provides an anaerobic and acidic environment for the growth of Firmicutes, which has acid hydrolytic or enzymatic hydrolytic functions (Ali et al., 2020; Xie et al., 2022). In the present study, the relative abundances of Firmicutes, Proteobacteria, Actinobacteria, and Bacteroidetes were influenced by ensiling days (*p* < 0.01), while the effect of wilting treatment on the relative abundances of all the bacterial phyla was not significant (*p* > 0.05) (Table 4), which indicated that ensiling duration is the crucial factor that primarily affects the changes in bacterial phyla.

**Table 4:** Significant analysis of wilting treatment, ensiling days, and their interaction on the relative abundances of main phyla and genera of sweet sorghum silage.

Item	Significance^a^		
	WT	ED	WT × ED
Phyla			
Firmicutes	NS	**	NS
Proteobacteria	NS	**	NS
Actinobacteriota	NS	***	NS
Bacteroidota	NS	***	*
Cyanobacteria	NS	NS	NS
Genera (Top 15)			
*Lactobacillus*	NS	**	NS
*Lactococcus*	*	***	NS
*Pantoea*	NS	**	NS
*Leuconostoc*	***	***	***
*unclassified_f__Enterobacteriaceae*	NS	NS	NS
*Enterobacter*	NS	NS	*
*Weissella*	*	*	NS
*unclassified_o__Lactobacillales*	NS	***	NS
*Enterococcus*	NS	***	*
*Klebsiella*	NS	NS	NS
*Pediococcus*	**	*	NS
*Serratia*	***	***	***
*Pseudomonas*	NS	***	**
*Microbacterium*	NS	***	NS
*Sphingomonas*	NS	***	NS

a WT, wilting treatment; ED, ensiling days; WT×ED, the interaction between wilting treatment and ensiling days. ‘*’, significant at *p* < 0.05; ‘**’, significant at *p* < 0.01; ‘***’, significant at *p* < 0.001; NS, not significant at *p* > 0.05.

To better understand the changes in the microbial communities, the relative abundances of the bacterial communities at the genus level are shown in Figure 5B. Among the unwilted preensiled sweet sorghum, the main bacterial genera were *Pantoea* (29.27%), *Pseudomonas* (12.66%), *Sphingomonas* (8.40%), *Microbacterium* (8.28%), and *Enterobacter* (4.53%), which have also been found in other plant materials, such as corn, alfalfa, and soybean (Ni et al., 2018; Nazar et al., 2020; Guo et al., 2021). Among the wilted preensiled sweet sorghum, the main bacterial genera were *Pantoea* (43.99%), *Microbacterium* (6.74%), *Sphingomonas* (6.37%), *Pseudomonas* (5.23%), *Klebsiella* (4.88%), and *Enterobacter* (3.28%). The composition of bacterial genera was not altered by the wilting treatment, and only the abundances of genera was affected, which is consistent with the results of Wang et al. (2019a).

After 3 days of ensiling, the relative abundance of *Pantoea* declined, while that of other genera, such as *Pseudomonas*, *Microbacterium*, and *Sphingomonas*, decreased to less than 1%. *Lactococcus* (22.68%) and *Leuconostoc* (23.26%) were the predominant bacterial genera in unwilted sweet sorghum silage, and *Lactococcus* (53.81) was the predominant bacterial genus in wilted sweet sorghum silage. *Lactococcus*, one of numerous genera in the lactic acid bacterium family, is a homofermenter that ferments carbohydrates, generating only one byproduct, LA (Nazar et al., 2020). Due to the high WSC content in the raw sweet sorghum material, coupled with its low buffering capacity, when anaerobic conditions occur, these WSCs are rapidly utilized by microorganisms such as *Lactococcus*, *Leuconostoc*, and *Enterobacter* to produce organic acids; subsequently, the pH of the silage rapidly decreases, thereby inhibiting undesirable microorganisms (Hu et al., 2009; Nazar et al., 2020).

With further extension of the ensiling time, the relative abundances of *Lactococcus*, *Leuconostoc*, *Enterobacter*, and *Enterococcus* decreased, while that of *Lactobacillus* gradually increased. A similar result was also reported in previous research; *Lactococcus*, *Leuconostoc,* and *Enterococcus* spp. commence ensiling fermentation and produce a suitable environment for the proliferation of *Lactobacillus* and are then replaced by more acid-tolerant lactobacilli, such as *Lactobacillus brevis* and *Lactobacillus plantarum* (Wang et al., 2019a; Ren et al., 2021). In the present study, the relative abundances of *Lactococcus*, *Weissella*, and *Pediococcus* were significantly influenced by wilting treatment (*p* < 0.05) (Table 4), which led to different fermentation patterns. It was demonstrated that the three most prevalent genera in silages are *Pediococcus*, *Weissella*, and *Lactobacillus* (Pang et al., 2011). Because *Weissella* and *Pediococcus* are less acid tolerant than *Lactobacillus*, they are typically regarded as early invaders during ensiling (Ni et al., 2017). The changes in these genera often affect the entire ensiling process. In this study, the relative abundance of *Lactobacillus* was 76.30 and 93.49% after 60 days of ensiling in wilted and unwilted sweet sorghum respectively, which may account for the relatively good fermentation quality. Therefore, sweet sorghum should be pretreated immediately after harvest. For good fermentation quality, moisture content should generally not exceed 800 g/kg fresh sample and storage should be at room temperature (approximately 25°C), with a recommended storage period of more than 30 days. Ensiling is an important method of preserving biomass material and extending its utilization. Different biomass materials have different characteristics, and their ensiling conditions were also different. Previous research has reported that the ensiing conditions for fresh rice straw are as follows: the temperature is 25 ± 3°C, the ensiling period is more than 60 days, and lactic acid bacteria and cellulase should also be added at the same time (Zhao et al., 2018). Similarly, for corn stove, ensiling is also a suitable pretreatment for subsequent bioenergy production, which requires additives, such as bacterial or enzymatic inoculants and is generally performed at a temperature of 25°C (Li et al., 2020). In the case of soybeans, it is necessary to add molasses in addition to lactic acid bacteria to improve fermentation quality during ensiling (Ni et al., 2017). Although our study showed that good fermentation quality could be achieved in high moisture sweet sorghum due to its high WSC and sufficient epiphytic lactic acid bacteria. However, screening of suitable additives to increase the biomass conversion efficiency and to improve the enzymatic efficiency also requires further research.

### 3.6 Correlations of bacterial communities with fermentation characteristics/carbohydrate components

**Figure 6:**
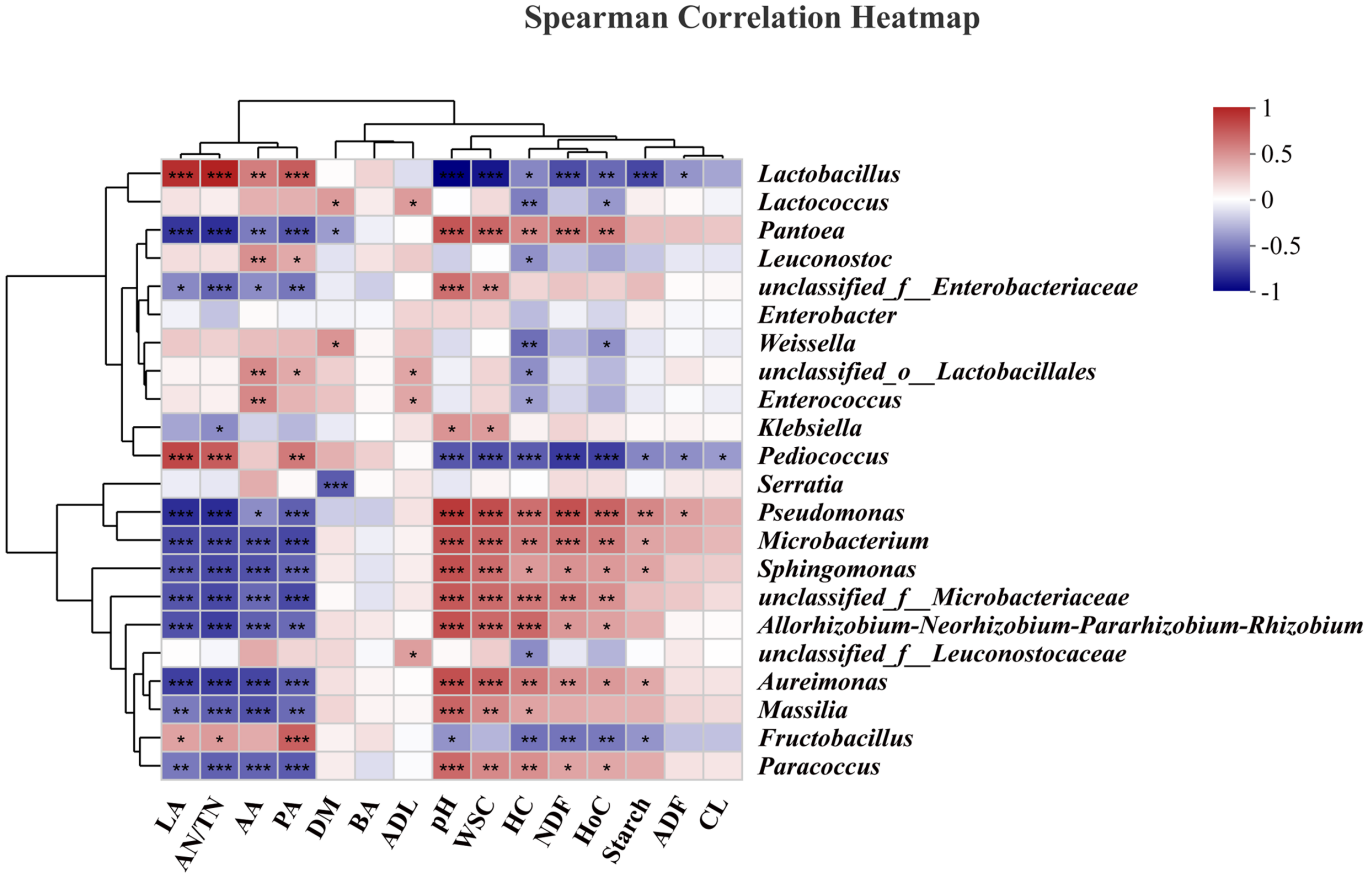
Correlation analysis of the bacterial community and fermentation characteristics/carbohydrate composition. LA, lactic acid; AN/TN, ammonia-nitrogen/total nitrogen; AA, acetic acid; PA, propionic acid; DM, dry matter; BA, butyric acid; ADL, acid detergent lignin; WSC, water-soluble carbohydrate; HC, hemicellulose, NDF, neutral detergent fiber; HoC, holocellulose; ADF, acid detergent fiber; CL, cellulose. “*”, significant at *p* < 0.05; “**”, significant at *p* < 0.01; “**”, significant at *p* < 0.001.

The correlations of the bacterial community with fermentation characteristics/carbohydrate components are shown in heatmaps (Figure 6). Specifically, *Lactobacillus* (*r* = 0.89, *p* < 0.001) and *Pediococcus* (*r* = 0.78, *p* < 0.001) were positively correlated with LA. *Lactobacillus* and *Pediococcus* are widely recognized for their significant roles in the production of LA during ensiling (Guo et al., 2023); our results also confirmed this phenomenon. *Lactobacillus* (*r* = 0.55, *p* < 0.01), *Leuconostoc* (*r* = 0.47, *p* < 0.01), and *Enterococcus* (*r* = 0.50, *p* < 0.01) were positively correlated with AA. *Lactobacillus* (*r* = 0.69, *p* < 0.001), *Leuconostoc* (*r* = 0.36, *p* < 0.05), *Pediococcus* (*r* = 0.57, *p* < 0.01), and *Fructobacillus* (*r* = 0.68, *p* < 0.001) were positively correlated with PA. These findings indicate that these bacteria utilize WSCs to generate AA and PA or are not inhibited by AA or PA. Considering the low relative abundances of the above genera, such as *Pediococcus* and *Fructobacillus* spp., this may be a common contributor to these correlations. Although AA can be further utilized, too much AA in silage is undesirable due to the greater associated biomethane loss than that with LA (Sun et al., 2021; Xie et al., 2022). *Lactobacillus* (*r* = −0.96, *p* < 0.001), *Pediococcus* (*r* = −0.64, *p* < 0.001), and *Fructobacillus* (*r* = −0.41, *p* < 0.05) were negatively correlated with pH, while *Pantoea* (*r* = 0.72, *p* < 0.001), *Pseudomonas* (*r* = 0.84, *p* < 0.001), *Microbacterium* (*r* = 0.73, *p* < 0.001), and *Sphingomona*s (*r* = 0.74, *p* < 0.001) were positively correlated with pH. The decrease in pH during the ensiling process could be due to the inhibition of aerobic genera (*Acinetobacter*, *Sphingomonas*, and *Pseudomonas* spp.) and the proliferation of lactic acid bacteria (*Lactobacillus* and *Pediococcus* spp.) (Zhao et al., 2023).

In this study, the WSC content was significantly negatively correlated with the abundances of *Lactobacillus* (*r* = −0.88, *p* < 0.001), and *Pediococcus* (*r* = −0.66, *p* < 0.001), which corresponded to changes in the LA content. In addition, for structural carbohydrates, *Lactobacillus* (*r* = −0.68, *p* < 0.001), *Pediococcus* (*r* = −0.75, *p* < 0.001), and *Fructobacillus* (*r* = −0.53, *p* < 0.01) were negatively correlated with NDF. *Lactobacillus* (*r* = −0.46, *p* < 0.05), *Lactococcus* (*r* = −0.49, *p* < 0.01), *Leuconostoc* (*r* = −0.42, *p* < 0.05), *Weissella* (*r* = −0.54, *p* < 0.01), *Enterococcus* (*r* = −0.36, *p* < 0.05), *Pediococcus* (*r* = −0.61, *p* < 0.001), and *Fructobacillus* (*r* = −0.54, *p* < 0.01) were negatively correlated with HC. HC is not as stable as CL and is less resistant to acidolysis and enzymolysis, and the acids produced during the ensiling process often led to hydrolysis of HC (Xie et al., 2022). Previous research has shown that HC undergoes extensive decomposition at lower pH values in silage (Dewar et al., 1963). *Lactobacillus*, *Enterococcus* are often classified as cellulolytic and xylan-degrading bacteria, and *Bacillus* and *Clostridium* are often classified as lignocellulolytic bacteria. In our study, such as *Lactobacillus*, *Lactococcus*, *Leuconostoc*, *Weissella*, *Enterococcuscan* can produce cellulase and xylanase, which degrade the CL and xylan in the feedstock, leading to changes in the structure of the biomass material (Ali et al., 2020; Xie et al., 2022). However, it should be noted that the ensiling process is a complex process of microbiological activity, and changes in the structure of the material often involve the interaction of multiple microorganisms. *Lactobacillus* (*r* = −0.41, *p* < 0.05) and *Pediococcus* (*r* = −0.42, *p* < 0.05) were negatively correlated with ADF, but *Pseudomonas* (*r* = 0.40, *p* < 0.05) was positively correlated with ADF. Moreover, *Pediococcus* (*r* = −0.39, *p* < 0.05) was negatively correlated with CL. The degradation of ADF and CL was limited to slight but constant acidolysis or depended on the addition of exogenous enzyme preparations or microbial preparations (Zhao et al., 2018).

## 4 Conclusion

This study revealed rapid and high-quality fermentation in wilted and unwilted sweet sorghum during ensiling, and significant changes in fermentation characteristics and carbohydrate components occurred during the initial 9 days of ensiling. The ensiling of sweet sorghum involved the degradation (5% ~ 10%) of NDF, and the degradation of NDF fit a first-order exponential decay model. A shift in *Lactococcus* dominance to *Lactobacillus* occurred before the first 9 days of ensiling, *Lactobacillus* was overwhelmingly dominant in wilted and unwilted sweet sorghum after ensiling for 60 days. Due to the higher WSC and sufficient epiphytic lactic acid bacteria of sweet sorghum raw material, the good fermentation quality can be obtained even under high moisture conditions. These results provide new insights into the efficiency of using sweet sorghum as a feedstock for biofuel production, and ensiling is proposed as a pretreatment for biofeedstock for subsequent biofuel production.

## Data Availability

The datasets presented in this study can be found in online repositories. The names of the repository/repositories and accession number(s) can be found at: https://www.ncbi.nlm.nih.gov/, PRJNA1068819.

## Footnotes

^1^https://cloud.majorbio.com
