# Supplementary material for: Dynamic changes in carbohydrate components and the bacterial community during the ensiling of wilted and unwilted sweet sorghum
Source: Front Microbiol. 2024 Aug 19;15:1452798. doi: 10.3389/fmicb.2024.1452798 (PMC11366713; doi:10.3389/fmicb.2024.1452798)
Supplement: Supplementary file 1 [file Table_1.DOCX]

Supplementary Material

# Supplementary Figures and Tables

## Supplementary Figures

| 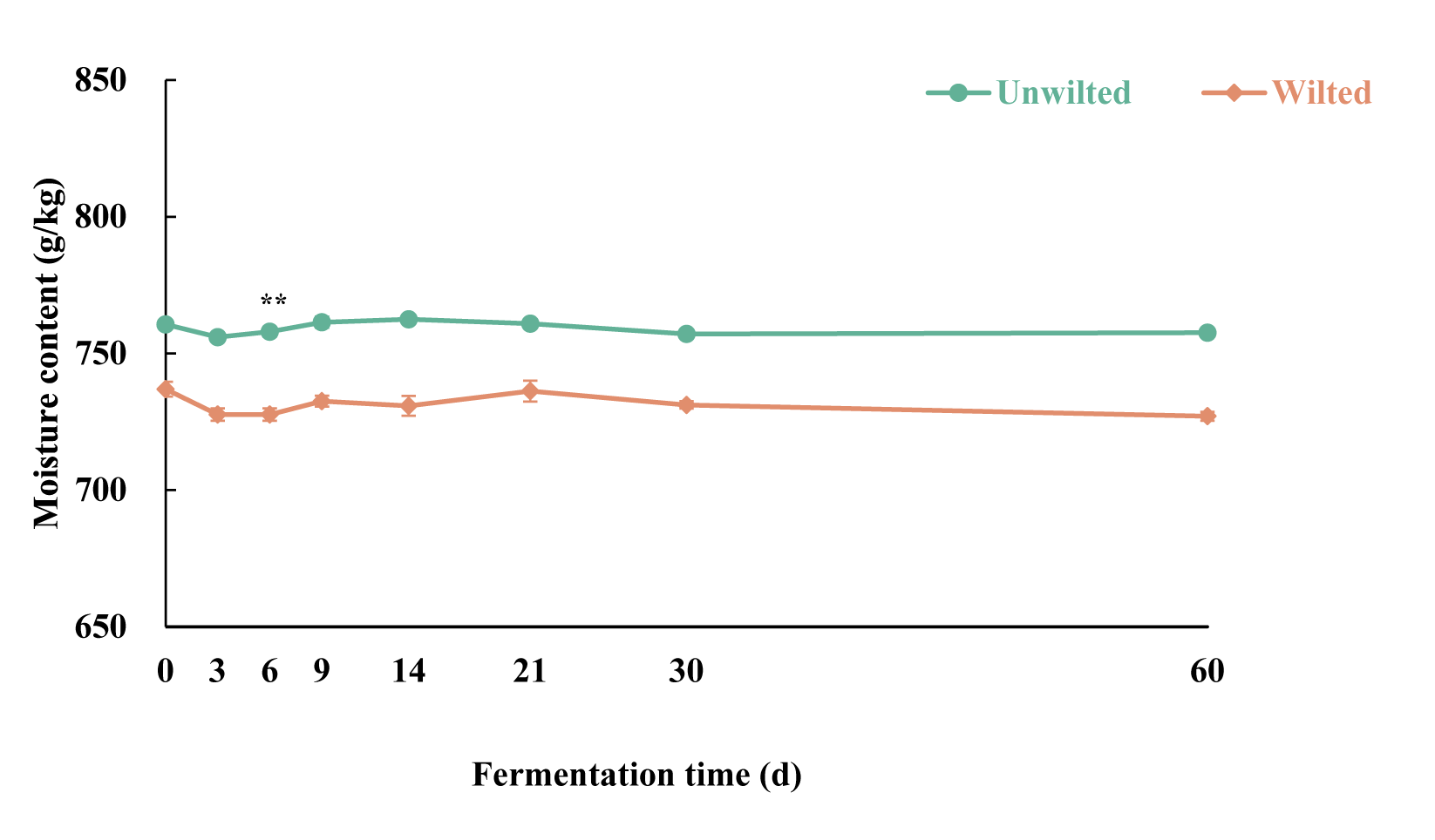 |
| --- |

**Supplementary Fig.1.** The dynamic changes in moisture content of wilted and unwilted sweet sorghum after 3, 6, 9, 14, 21, 30 and 60 days of ensiling. Asterisks indicate significant differences between different treatments on the same ensiling days (*, significant at *P*<0.05, and **, significant at *P*<0.01).

| 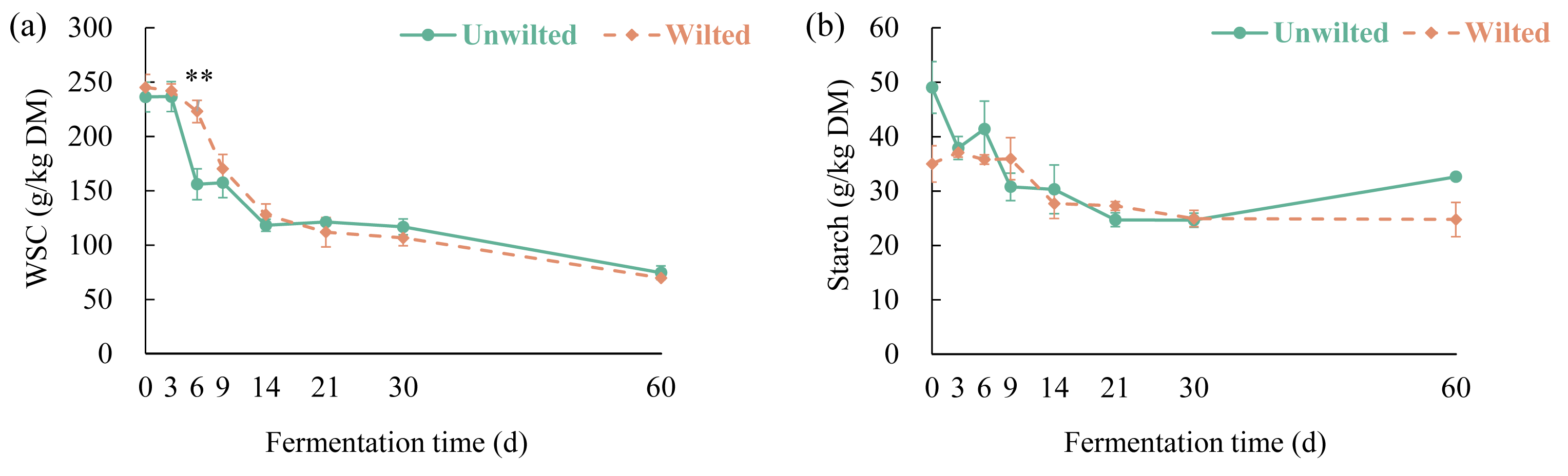 |
| --- |

**Supplementary Fig.2.** The dynamic changes in (a) water-soluble carbohydrate (WSC) and (b) starch in wilted and unwilted sweet sorghum after 3, 6, 9, 14, 21, 30 and 60 days of ensiling. DM, dry matter. Asterisks indicate significant differences between different treatments on the same days of ensiling (*, significant at *P*<0.05, and **, significant at *P*<0.01).
